# Use of data mining surveillance system in real time detection and analysis for healthcare-associated infections

**DOI:** 10.1186/1753-6561-5-S6-P235

**Published:** 2011-06-29

**Authors:** CM Ke, FJ Huang, SS Lee, YS Chen, PJ Hsieh, YE Lin

**Affiliations:** 1 Kaohsiung Veterans General Hospital, Kaohsiung, Taiwan, China; 2 National Kaohsiung Normal University, Kaohsiung, Taiwan, China

## Introduction / objectives

Hospital information system have been developed to provide better quality of patient care and efficient hospital management. However, these data have not been linked to infection control surveillance. Often time infection control practitioners (ICPs) can only detect healthcare-associated infections (HAIs) based on retrospective analysis of lab results and patient data. Thanks to data mining and artificial intelligence software, the ICPs may be able to detect HAIs in real time based on lab results in certain infections. Thus, the objective is to use knowledge management and artificial intelligence to design a real time HAI surveillance system.

## Methods

HAIs caused by multi-drug resistant organisms are our targets in a medical center in southern Taiwan. We designed an automated mechanism to import laboratory results combined with patient-specific data (DOA, bed #, lab orders, etc..). The moving average and trend of positive cultures were plotted. We also used data mining rules (Apriori, Anomaly, and Time-Series analysis) to determine the potential of undetected HAI and outbreaks.

## Results

The moving average is a good tool of predicting carbapenem-resistant *A. baumannii* (CRAB) transmission in ICUs. Our surveillance may also determine the potential index patient in a time-series analysis. The Anomaly analysis was able to point out the potential patient wards to have an outbreak by detecting multi-drug resistant organisms (eg. CRAB and MRSA) or rare organisms (eg. VRE) from laboratory results.

## Conclusion

Our results showed that a real time rule-based automated infection surveillance system is possible to assist ICPs to detect potential HAIs which saves time and manpower to prevent nosocomial infections.

## Disclosure of interest

None declared.

